# The Role of Basement Membranes in Cerebral Amyloid Angiopathy

**DOI:** 10.3389/fphys.2020.601320

**Published:** 2020-11-25

**Authors:** Matthew D. Howe, Louise D. McCullough, Akihiko Urayama

**Affiliations:** Department of Neurology, McGovern Medical School, The University of Texas Health Science Center at Houston, Houston, TX, United States

**Keywords:** CAA, basement membrane, amyloid-beta, perivascular transport, dementia

## Abstract

Dementia is a neuropsychiatric syndrome characterized by cognitive decline in multiple domains, often leading to functional impairment in activities of daily living, disability, and death. The most common causes of age-related progressive dementia include Alzheimer’s disease (AD) and vascular cognitive impairment (VCI), however, mixed disease pathologies commonly occur, as epitomized by a type of small vessel pathology called cerebral amyloid angiopathy (CAA). In CAA patients, the small vessels of the brain become hardened and vulnerable to rupture, leading to impaired neurovascular coupling, multiple microhemorrhage, microinfarction, neurological emergencies, and cognitive decline across multiple functional domains. While the pathogenesis of CAA is not well understood, it has long been thought to be initiated in thickened basement membrane (BM) segments, which contain abnormal protein deposits and amyloid-β (Aβ). Recent advances in our understanding of CAA pathogenesis link BM remodeling to functional impairment of perivascular transport pathways that are key to removing Aβ from the brain. Dysregulation of this process may drive CAA pathogenesis and provides an important link between vascular risk factors and disease phenotype. The present review summarizes how the structure and composition of the BM allows for perivascular transport pathways to operate in the healthy brain, and then outlines multiple mechanisms by which specific dementia risk factors may promote dysfunction of perivascular transport pathways and increase Aβ deposition during CAA pathogenesis. A better understanding of how BM remodeling alters perivascular transport could lead to novel diagnostic and therapeutic strategies for CAA patients.

## Introduction

Alzheimer’s disease (AD) and vascular cognitive impairment (VCI) nominally represent the most common forms of age-related progressive dementia, and multiple studies have demonstrated that mixed vascular and AD-type amyloid-β (Aβ) pathology is a common finding on autopsy in elderly dementia patients (Jellinger, 2002; White et al., 2005; Schneider et al., 2007). This may be partially explained by epidemiological studies which show that the majority of risk factors for both types of dementia are related to cerebrovascular disease and include APOE-ε4 genotype, advanced age, hypertension, hyperlipidemia, diabetes, obesity, and smoking (Corder et al., 1993; Kivipelto et al., 2006; Gorelick et al., 2011; Viticchi et al., 2015; Santos et al., 2017; Vos et al., 2017; Deckers et al., 2019). These shared risk factors suggest that an interaction between cerebrovascular disease and Aβ deposition exists, and may help to explain the relatively high incidence of mixed dementia (Deramecourt et al., 2012). While a causal link has yet to be established, a growing body of clinical and pre-clinical evidence suggests that cerebrovascular disease can be a major driver of Aβ deposition and cognitive decline in patients suffering from age-related progressive dementia (Santos et al., 2017).

Cerebral amyloid angiopathy (CAA), which refers to the presence of Aβ plaques in the walls of cerebral vessels, is associated with significant morbidity and mortality in afflicted individuals (Vonsattel et al., 1991; Greenberg and Vonsattel, 1997; Greenberg et al., 2020). CAA affects approximately 82–98% of AD patients on autopsy and is associated with an increased burden of cerebrovascular insults (Jellinger, 2002; Attems and Jellinger, 2014). Neuropathological studies demonstrate that CAA preferentially affects the outer leptomeningeal vessels on the surface of the brain, and can also spread to involve more distal intraparenchymal arteries, arterioles, and capillaries in some patients (Thal et al., 2002; Yamada, 2015; Greenberg et al., 2020). CAA pathology is identified by the formation of small Aβ deposits within the basement membrane (BM), which is a thin perivascular layer of extracellular matrix (ECM) that plays an important role in maintaining the integrity of the blood-brain barrier (Yamaguchi et al., 1992; Reed et al., 2019; Gireud-Goss et al., 2020). As BM remodeling and Aβ deposition progress, CAA impairs cognitive function via multiple mechanisms, including reduced neurovascular coupling, progressive vasculopathy as well as neurological emergencies such as lobar intracerebral hemorrhage.

The pathophysiology of sporadic, age-related CAA is multifactorial and may be driven, in part, by disruption of perivascular Aβ transport along with the remodeled BM. Recent advances in the field have shed light on how specific, early changes in BM morphology and composition may impair the perivascular transport of Aβ. In the present review, we will first describe the anatomy, morphology, molecular composition, and physiology that support perivascular transport through healthy BM. We will then explore how these processes are altered in individuals at-risk of or affected by CAA. Finally, we will highlight multiple potential mechanisms for how BM remodeling could impair perivascular transport and drive CAA pathogenesis. Our hope is that this review will highlight the common molecular pathophysiology of conditions that lead to impaired perivascular transport and early Aβ deposition, which could have major implications for the prevention, early detection and treatment of CAA.

## Changes in BM Morphology and Composition During CAA Pathogenesis

### Anatomical Overview of the Healthy Perivascular Space

**FIGURE 1:**
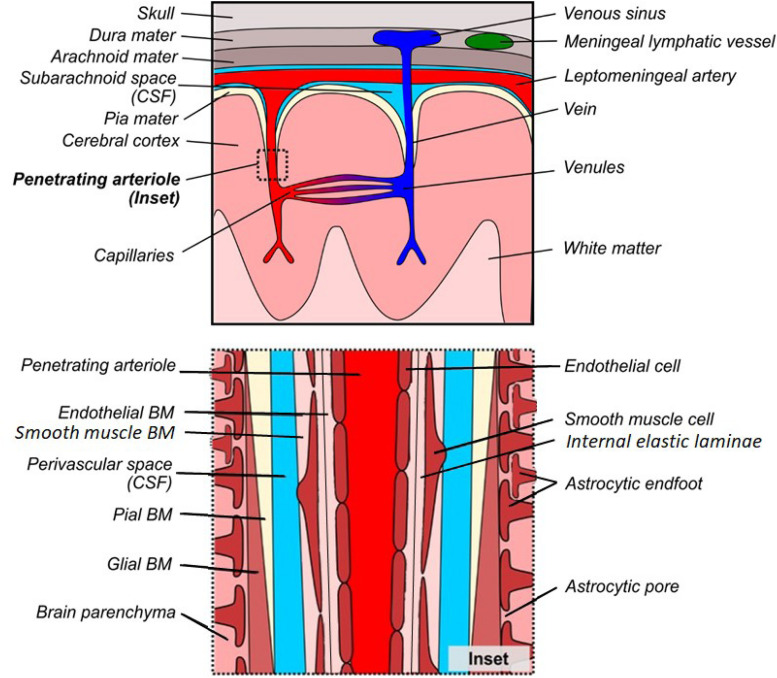
Gross and microscopic anatomy of the perivascular space. **Top:** Gross anatomy of meninges, cortical vessels and associated perivascular networks. **Bottom:** Microscopic anatomy of the perivascular space surrounding a penetrating arteriole in normal cortex. There is debate as to whether the perivascular space is truly distinct from the glial/pial and endothelial BMs, however it has been drawn separately to facilitate understanding of the various models of perivascular transport.

The brain contains a dense network of blood vessels and perivascular spaces that act in concert to deliver oxygen and nutrients to the parenchymal tissue, as well as remove waste products. This network can be conceptualized as the cerebrovascular tree (Chandra et al., 2017; Kirst et al., 2020; Figure 1). Briefly, the internal carotid and vertebral arteries converge upon the Circle of Willis within the cerebrospinal fluid (CSF)-containing subarachnoid space (SAS) at the base of the skull (Chandra et al., 2017; Kirst et al., 2020). These vessels, along with the SAS, further subdivide and extend around the brain, creating a tortuous network of surface vessels surrounded by large perivascular spaces, called cisterns, that bathe the outside of the brainstem, cerebellum, and cerebral cortex in CSF (Adeeb et al., 2013). These large and medium-sized surface vessels eventually subdivide into smaller leptomeningeal arteries that penetrate the pia mater through Virchow-Robin space, which is a CSF-filled perivascular invagination of cortical tissue that is continuous with the surface cisterns (Shih et al., 2015; Morris et al., 2016; Nakada et al., 2017; Hannocks et al., 2018; Pizzo et al., 2018). As the leptomeningeal arteries pass through Virchow-Robin space, they branch into penetrating arterioles and capillary beds, which subsequently drain into venules, veins, and venous sinuses (Kiliç and Akakin, 2008; Marín-Padilla and Knopman, 2011; Shih et al., 2015). The perivascular space (PVS) continues alongside leptomeningeal arteries and penetrating arterioles, providing an important channel that bathes the cerebral vasculature in CSF and promotes the perivascular clearance of waste products from the brain parenchyma (Alcolado et al., 1988; Zhang et al., 1990; Iliff et al., 2012, 2013a,b; Xie et al., 2013). While the nature of the PVS at the capillary vasculature remains elusive, compelling evidence suggests that the vascular BM serves as a canal for solute clearance (Morris et al., 2016). These important clearance pathways are dependent on a complex interplay between the cells, extracellular BM proteins, and the associated PVSs that together form a unique microenvironment.

Under normal conditions, the healthy PVS consists of three distinct cellular layers that are associated with BM proteins (Figure 1, inset). In terms of cellular components, the astrocytic endfeet surround the intracerebral vessels and define the outermost layer of the perivascular tissue, with an additional contribution of microglial processes (Mathiisen et al., 2010; Joost et al., 2019). Additional cell types, including mural cells [referring to vascular smooth muscle cells (VSMCs), pericytes and fibroblasts] as well as perivascular macrophages, comprise the loosely defined middle cellular layer and are embedded within the BM (Goldmann et al., 2016; Nikolakopoulou et al., 2017; Chasseigneaux et al., 2018; Vanlandewijck et al., 2018). Finally, the endothelial cells form a continuous barrier, yet the interface between the CNS and the periphery, which comprises the innermost layer of the perivascular tissue (Ben-Zvi et al., 2014; Banks, 2016). In addition to these separate cellular layers, the PVS includes two major, extracellular components: the protein-rich BM, and the fluid-filled PVS. The composition of the BM is dependent on the cell types contained within each region of the perivasculature and can be divided into anatomically and functionally distinct endothelial, pial, and glial layers (Hannocks et al., 2018). The innermost endothelial layer of the BM is directly apposed to the abluminal surface of the endothelial cell, and extends outward to the PVS (Sapsford et al., 1983; Abbott et al., 2018). On the opposite side of the PVS, the outer BM is further subdivided into pial and glial layers. The pial layer of the BM is present along the outer surface of the PVS of leptomeningeal arteries and is produced by meningeal epithelial cells and highly attenuated layers of astrocytic endfoot forming the glia limitans (Alcolado et al., 1988; Zhang et al., 1990; Sofroniew, 2015). The glial layer of the BM replaces the pial layer along penetrating arteries and arterioles. This layer is secreted by the perivascular endfeet of astrocytes and forms the prominent outer border of the PVS in arterioles, which eventually merges with the endothelial layer to form a thin capillary BM (Hauw et al., 1975; Watanabe et al., 2010). This separation of layers re-emerges in the post-capillary venule and continues to form a paravenous/extramural perivascular drainage system (Iliff et al., 2012; Hladky and Barrand, 2018).

### Composition and Function of the Healthy BM

During development, endothelial cells, astrocytes, and pericytes produce the major components of their respective BM partitions (Chouchkov et al., 1987). In the healthy adult brain, the BM is comprised of large complexes of ECM molecules, including collagen IV, nidogen-1 and -2, various heparan sulfate proteoglycans (HSPGs), and region-dependent laminin isoforms (Engelhardt and Sorokin, 2009; Table 1). The BM plays an important role in providing structural support and regulating the activity of the cellular components in the PVS (Thomsen et al., 2017b). The BM regulates local cellular function by binding to integrin receptors, which are a family of cell membrane receptors that contain both α and β subunits. Different classes of integrins are expressed by endothelial cells (α1β1, α3β, α6β1, αvβ1) (Paulus et al., 1993; Milner and Campbell, 2002, 2006; Engelhardt and Sorokin, 2009), pericytes (α4β1) (Grazioli et al., 2006) and astrocytes (α1β1, α5β1, α6β1) (Milner and Campbell, 2006). In addition to their role in maintaining the structure of the PVS, many integrins also transduce signals from surrounding BM proteins, alerting cells to changes in their extracellular environment. For example, α5β1 integrin, which is expressed on the outer surface of astrocytic endfeet, binds to fibronectin within the BM and regulates downstream signaling pathways to promote cellular adhesion and angiogenesis during tissue development and after injury (Wang and Milner, 2006; Yanqing et al., 2006; Mahalingam et al., 2007; Huveneers et al., 2008; Wang et al., 2010; Hielscher et al., 2016; Lee et al., 2019).

**TABLE 1:** BM proteins expressed in mature intraparenchymal and meningeal vessels.

	Perivascular Localization	Cellular source
**Collagens**		
Collagen IV	• Brain microvessels (Urabe et al., 2002) • Choroid plexus (Urabe et al., 2002) • Leptomeningeal arteries (Keable et al., 2016)	• Endothelial cells (Jeanne et al., 2015) • Meningeal cells (Sievers et al., 1994) • Pericytes (Jeanne et al., 2015)
**Fibulins**		
Fibulin-1	• Brain microvessels (Rauch et al., 2005) • Choroid plexus (Rauch et al., 2005)	• Endothelial cells (Thomsen et al., 2017a)
Fibulin-2	• Larger intraparenchymal vessels (Rauch et al., 2005)	• Endothelial cells (Thomsen et al., 2017a)
Fibulin-5	• Brain microvessels (Guo et al., 2016)	• Astrocytes (Howe et al., 2019) • Endothelial cells (Albig and Schiemann, 2004)
**Heparan sulfate proteoglycans**		
Perlecan	• Brain microvessels (Endothelial > Glial BM) (Agrawal et al., 2006; Gustafsson et al., 2013)	• Astrocytes (Howe et al., 2019) • Endothelial cells (Thomsen et al., 2017a)
Agrin	• Brain microvessels (Glial > Endothelial BM) (Barber and Lieth, 1997; Agrawal et al., 2006; Wolburg-Buchholz et al., 2009)	• Astrocytes (Howe et al., 2019; Noël et al., 2019) • Endothelial cells (Thomsen et al., 2017a)
Collagen XVIII	• Brain microvessels (Utriainen et al., 2004) • Choroid plexus (Utriainen et al., 2004) • Leptomeningeal arteries (Utriainen et al., 2004)	• Astrocytes (Howe et al., 2019) • Endothelial cells (Thomsen et al., 2017a)
**Laminins**		
Laminin 111	• Brain microvessels (parenchymal BM) (Jucker et al., 1996; Sixt et al., 2001; Halder et al., 2018; Hannocks et al., 2018)	• Astrocytes (Sixt et al., 2001)
Laminin 211	• Brain microvessels (parenchymal BM) (Jucker et al., 1996; Sixt et al., 2001; Hannocks et al., 2018)	• Astrocytes (Menezes et al., 2014)
Laminin 411	• Brain microvessels (endothelial BM) (Sixt et al., 2001; Halder et al., 2018)	• Endothelial cells (Frieser et al., 1997; Thomsen et al., 2017a)
Laminin 421	• Brain microvessels (endothelial BM) (Sixt et al., 2001; Ljubimova et al., 2006)	• Endothelial cells (Ljubimova et al., 2006)
Laminin 511	• Brain microvessels (endothelial BM) (Sixt et al., 2001)	• Endothelial cells (Thomsen et al., 2017a)
**Nidogens**		
Nidogen-1	• Brain microvessels (Niquet and Represa, 1996)	• Astrocytes (Grimpe et al., 1999) • Endothelial cells (Thomsen et al., 2017a) • Meningeal cells (Sievers et al., 1994)
Nidogen-2	• Brain microvessels (Keable et al., 2016)	• Endothelial cells (Thomsen et al., 2017a)
**Other glycoproteins**		
Fibronectin	• Brain microvessels (Keable et al., 2016; Andrews et al., 2018; Howe et al., 2018a, 2019)	• Astrocytes (Howe et al., 2019) • Endothelial cells (Thomsen et al., 2017a; Andrews et al., 2018) • Meningeal cells (Sievers et al., 1994) • VSMCs (Andrews et al., 2018)

Together, the ECM molecules and cellular components at the PVS support tissue homeostasis via a variety of functions in the healthy brain, including maintaining the structural integrity of vessels and permitting the exchange of fluids and solutes between the PVS and the brain parenchyma. In the next section, we will take a detailed look at the molecular and cellular changes that occur within the BM during CAA pathogenesis.

### BM Remodeling During CAA Pathogenesis

**FIGURE 2:**
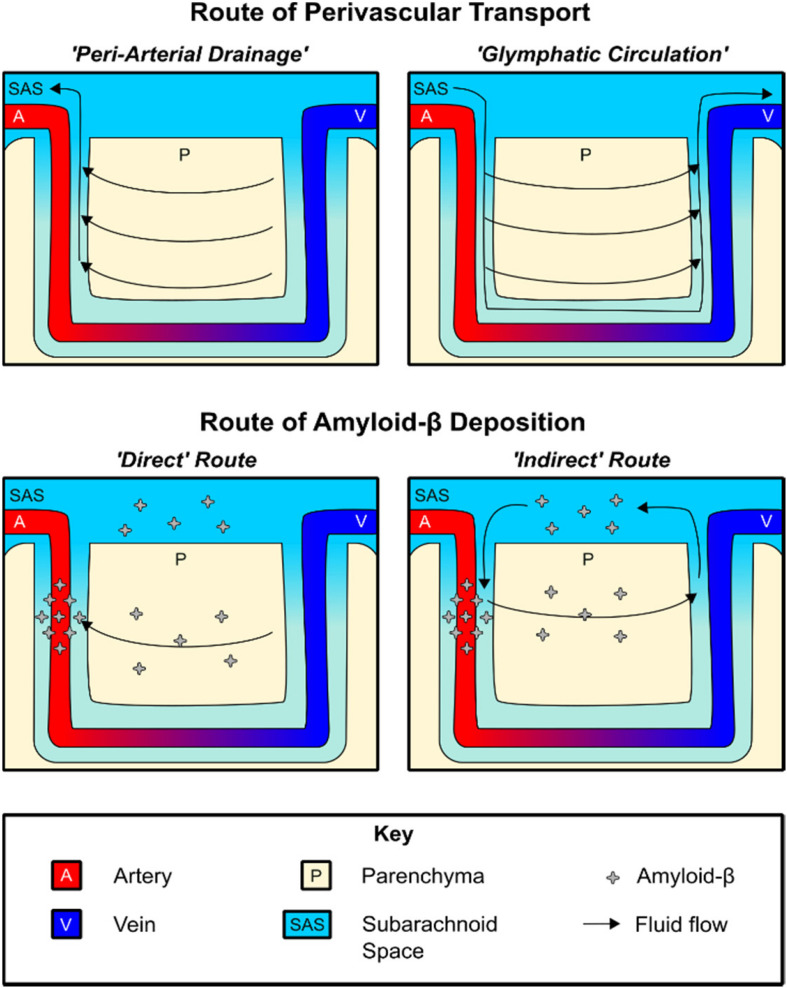
Routes of perivascular fluid transport and Aβ deposition. **Top:** Diagram of perivascular fluid transport under the peri-arterial drainage and glymphatic circulation models, respectively. **Bottom:** Both direct and indirect routes of Aβ deposition may contribute to CAA pathology.

The pathogenesis of sporadic CAA and AD overlap each other (Greenberg et al., 2020), and this interaction between these two diseases may be centered at the PVS. CAA is associated with profound changes in BM morphology and composition (Okoye and Watanabe, 1982; Snow et al., 1988, 1994; Perlmutter et al., 1990, 1991; Su et al., 1992; Shimizu et al., 2009), which may predispose vessels to the development of Aβ deposits. Table 2 summarizes the changes in BM protein composition in AD patients as a clinically relevant proxy to CAA as well as APP overexpression animal models which exhibit CAA pathology. Multiple neuropathological studies in human patients and animal models support the hypothesis that BM remodeling sensitizes the perivascular tissue to the development of CAA. Yamaguchi et al. (1992) identified the pial BM layer of large leptomeningeal arteries, as well as the glial BM of small leptomeningeal arteries and penetrating cortical arterioles, as the initial sites of amyloid deposition (Figures 1, 2). The study found that, in sites with more advanced pathology, larger amyloid deposits were found to extend into the endothelial BM, and were associated with mural cell degeneration. Smaller amyloid deposits were also identified in the capillary beds of some patients, and were associated with abnormal thickening of the capillary BM (Yamaguchi et al., 1992). Similar findings were reported in a subsequent study, which identified two distinct subtypes of CAA with and without capillary involvement (Thal et al., 2002). BM thickening and degeneration, abnormal HSPG deposits, and irregular vessel thickness have been noted in multiple studies of AD and CAA (Okoye and Watanabe, 1982; Snow et al., 1988, 1994; Perlmutter et al., 1990, 1991; Su et al., 1992; Shimizu et al., 2009). Specific changes in BM composition generally include increased collagen IV, fibronectin, agrin, and perlecan expression (Table 2). In addition to these molecular changes in BM composition and morphology, multiple cell types associated with the cerebral vasculature exhibit altered morphology and function in CAA. Several studies have found that Aβ pathology was associated with loss of endothelial cells and disruption of blood–brain barrier integrity (De Jager et al., 2013; Magaki et al., 2018), degeneration of mural cells (Yamaguchi et al., 1992; Tian et al., 2006), impairment of pericyte function by oligomeric Aβ (Nortley et al., 2019), as well as increased reactive astrocytosis with dystrophic endfeet surrounding BM amyloid deposits (Giannoni et al., 2016; Yang et al., 2017). Overall, these studies support the claim that CAA is associated with widespread changes to the PVS, including BM composition and cellular morphology.

**TABLE 2:** Changes in BM composition in AD patients and transgenic mouse models compared to age-matched controls.

References	Subjects	Collagen IV	Fibronectin	Agrin	Perlecan	Laminin	Nidogen-2
Kalaria and Pax (1995)	AD patients	↑	–	–	–	–	–
Berzin et al. (2000)	AD patients	–	–	↑	–	–	–
Farkas et al. (2000)	AD patients	↑	–	–	–	–	–
Keable et al. (2016)	AD patients	↔	↔	–	–	–	↓
Lepelletier et al. (2017)	AD Patients	↑	↑	–	↑	–	–
Magaki et al. (2018)	AD patients*	↑	–	–	–	–	–
Singh-Bains et al. (2019)	AD patients	↔	↑	–	–	–	–
Bourasset et al. (2009)	Transgenic mice (3xTg)	↑	–	–	–	–	–
Gama Sosa et al. (2010)	Transgenic mice (P117L)**	↔	↔	–	–	↔	–
Kurata et al. (2011)	Transgenic mice (Tg2576)	↓	–	–	–	–	–
Merlini et al. (2011)	Transgenic mice (Tg-ArcAβ)	–	–	–	–	↑	–
Mehta et al. (2013)	Transgenic mice (3xTg)	↑	–	–	–	–	–

Arrows denote increased, decreased or no significant difference in brain expression relative to controls.*Significantly increased collagen IV was observed in AD patients without CAA, with a trending increase in AD patients with CAA.**P117L mice did not exhibit amyloid pathology.

The pathophysiologic relationship between BM remodeling and Aβ deposition is becoming clearer, as recent advances in our understanding of perivascular amyloid transport have identified fundamental links between these two processes. In the following section, we will explore the evidence for the existence of a perivascular fluid transport system in the brain, and how dysregulation of this system contributes to CAA. With this knowledge in hand, we will then conclude by revisiting how the above changes in BM composition could regulate changes in perivascular transport during CAA pathogenesis.

## Perivascular Transport Networks in the Healthy and Diseased Brain

### Perivascular Transport of Solutes in the Brain

The brain extracellular space (ECS) is filled with extracellular matrix components attached to the cellular geometry, forming a multitude of channels where interstitial fluid (ISF) and solutes are transported. The ISF contains secreted Aβ_1__–__4__0_ and provides a route for its removal, which is potentially subject to a collection of hydrostatic forces that are generally referred to as “bulk flow,” however, it seems such directional flow is confined to the PVSs in the brain (Syková and Nicholson, 2008). Compelling evidence suggests that brain parenchymal solute distribution is predominantly mediated by the thermodynamic free diffusion process through the ECS. *In vivo* visualization of the free diffusion process was established by a series of studies by Nicholson and colleagues (Nicholson and Tao, 1993; Thorne et al., 2004, 2008; Thorne and Nicholson, 2006), which recorded the symmetric diffusion pattern of tracers in the brain.

Earlier studies found that bulk flow may depend on hydrostatic pressure gradients within the brain parenchyma that drive the removal of solutes along with ISF movement. Bulk flow of ISF has been estimated to be relatively fast, occurring at a rate of ∼10.5 μm/min in healthy brain tissue (Rosenberg et al., 1980). Through these studies, the directional fluid movement and the diffusive ECS transport in the brain parenchyma may be controversial, yet the existence of bulk flow was clearly visible in the PVS in an early experiment as well as a recent particle tracking study (Ichimura et al., 1991; Mestre et al., 2018b). While these differing mechanisms for solute translocation require further study, the consensus is that parenchymal solute clearance occurs. Such clearance pathways may be grouped into two general models of perivascular transport, as described below.

The first model, which we will refer to as “peri-arterial drainage,” proposes that ISF transport occurs along the outer BM of cerebral arteries (Morris et al., 2014; Figure 2). This model is based on a series of studies showing that intraparenchymally injected fluorescent tracers rapidly migrate to the glial BM of capillaries and arterioles, and were transported opposite blood flow to the pial BM surrounding leptomeningeal arteries (Carare et al., 2008; Arbel-Ornath et al., 2013). Additional work found that intraparenchymally injected fluorescent Aβ_1__–__4__0_ also distributed along with the BM of capillaries, arterioles, and leptomeningeal arteries, depositing within the vessel wall in a distribution similar to that seen in CAA patients (Hawkes et al., 2011, 2012, 2013, 2015; Morris et al., 2016). A recent study found that, in healthy brain tissue, CSF influx is proposed to take place along the pial/glial BM while ISF efflux occurs along with the smooth muscle BM (tunica media) (Albargothy et al., 2018).

The second model, sometimes referred to as “glial-lymphatic (glymphatic) circulation,” posits the existence of circulation of CSF within the PVS surrounding cerebral blood vessels, which we will specifically refer to as *para-*vascular influx/efflux. In this model, para-arterial CSF influx is postulated to drive the clearance of Aβ_1__–__4__0_ and other solutes along para-venous routes (Iliff et al., 2012; Jessen et al., 2015), where it is returned to the SAS for transport along multiple pathways, including the recently described meningeal lymphatics (Louveau et al., 2015, 2017; Plog et al., 2015).

Regardless of the driving kinetic model, perivascular Aβ_1__–__4__0_ transport provides an intriguing explanation for the arterial distribution of amyloid plaque pathology and also explains why pathology first occurs within the BM (Yamaguchi et al., 1992; Thal et al., 2002; Morris et al., 2014; Keable et al., 2016). In terms of understanding the pathogenesis of CAA, both peri-arterial drainage and glymphatic circulation describe potential mechanisms by which Aβ_1__–__4__0_ deposition occurs on the vascular wall. This deposition may be conceptualized as occurring both “directly” and “indirectly.” The peri-arterial drainage model provides a “direct” pathway by which Aβ_1__–__4__0_ produced by neurons is rapidly deposited within the arterial BM during CAA pathogenesis as a result of impaired clearance from the brain (Morris et al., 2014; Keable et al., 2016). On the other hand, a novel meningeal contribution to the pathogenesis of CAA may be derived from the glymphatic circulation model, in which CSF Aβ_1__–__4__0_ may pathologically re-enter the brain along para-arterial influx routes and become deposited along with a similar distribution in an “indirect” manner. In support of the latter model, Peng et al. (2016) found that CSF-derived Aβ_1__–__4__0_ colocalizes with existing CAA, and that high levels of CSF Aβ_1__–__4__0_ can slow the rate of perivascular transport. The importance of this route may further increase in the aged brain, as meningeal drainage pathways become less efficient and could theoretically favor re-circulation of CSF Aβ_1__–__4__0_ (Louveau et al., 2015, 2017; Plog et al., 2015; Da Mesquita et al., 2018; Ma et al., 2019). This indirect route of deposition may explain why lower CSF Aβ_1__–__4__0_ levels have been found to be a biomarker of CAA, as declining CSF Aβ_1__–__4__0_ levels may reflect a disequilibrium between para-venous clearance and para-arterial deposition (Charidimou et al., 2018).

In addition to the above experimental work, both peri-arterial drainage and glymphatic clearance system are equipped with theoretical considerations. The feasibility of the peri-arterial drainage model was estimated by mathematical models, showing that cerebral arterial pulsations, generated by VSMCs, may produce sufficient reflected waves within the BM that drive bulk flow along periarterial vascular routes (Schley et al., 2006; Wang and Olbricht, 2011; Coloma et al., 2016; Aldea et al., 2019). However, several computational studies predicted that the high degree of hydraulic resistances within the BM theoretically limits the distance of ISF transport (Asgari et al., 2015; Faghih and Sharp, 2018; Ray et al., 2019). Also, there is a possibility that that CSF tracer translocation to BM might be associated with spreading depolarizations and tissue processing artifacts as opposed to physiological transport (Bakker et al., 2016; Schain et al., 2017; Mestre et al., 2018b, 2020).

While Faghih and Sharp (2018) also suggested the glymphatic circulation is implausible, this has been rebutted by another study finding that the degree of hydraulic resistance may be overestimated in some models (Tithof et al., 2019), as well as another study that found the diameter of the PVS may be underestimated in other studies due to shrinkage that occurs with tissue fixation (Mestre et al., 2018b). Overall, while mathematical models provide a conflicting picture, the experimental data support a role for glymphatic flow in driving waste clearance under a variety of physiological conditions. Importantly, both peri-arterial efflux and glymphatic circulation may contribute to the pathophysiology of CAA, and these pathways are likely not mutually exclusive from one another.

## Perivascular Transport Is Altered by Aging and Other Disease States

Multiple vascular risk factors are associated with CAA, including aging, ApoE-ε4 genotype, cerebrovascular disease, hyperlipidemia, and hypertension. These factors involve changes in the BM which may affect perivascular transport and amyloid clearance as summarized in Table 3.

**TABLE 3:** Studies of perivascular transport in animal models of AD and related dementia risk factors.

References	Subjects	Experimental group	Control group	Tracer influx	Tracer efflux	Aβ deposition	Aβ clearance
**CAA**							
Hawkes et al. (2011)	Transgenic mice	*Tg2576*	Wild-type	–	↓,↑*	–	–
Peng et al. (2016)	Transgenic mice	*APP/PS1*	Wild-type	↓	↓	↑	↓
van Veluw et al. (2019)	Transgenic mice	*APP/PS1*	Wild-type	–	↓	–	–
**Aging**							
Hawkes et al. (2011)	Wild-type mice	Aged (22 months-old)	Young (3 months-old)	–	↓	–	–
Hawkes et al. (2013)	Wild-type mice	Aged (22 months-old)	Young (2 months-old)	–	–	–	↓
Kress et al. (2014)	Wild-type mice	Aged (18 months-old)	Young (2 months-old)	↓	↓	–	↓
Da Mesquita et al. (2018)	Wild-type mice	Aged (22 months-old)	Young (2 months-old)	↓	↓	–	–
**ApoE-ε4 genotype**							
Hawkes et al. (2012)	Transgenic mice	*Hu-APOE4*	*Hu-APOE2*	–	–	↑	–
Achariyar et al. (2016)	Wild-type mice	CSF Hu-APOE4**	CSF Hu-APOE2/3	↓	–	–	–
**Cerebrovascular disease**							
Gaberel et al. (2014)	Wild-type mice	Stroke (SAH, tMCAO)	Sham	↓	–	–	–
Wang et al. (2017)	Wild-type mice	Stroke (MMI)	Sham	↓	–	–	–
Howe et al. (2018a)	Wild-type mice	Aged stroke (pDMCAO)	Aged sham	↓	–	↑	–
Howe et al. (2019)	Wild-type mice	Stroke (pDMCAO)	Sham	↓	–	–	–
**Hyperlipidemia**							
Hawkes et al. (2015)	Wild-type mice	High-fat diet	Standard diet	–	–	↑	↓
Ren et al. (2017)	Transgenic mice	*Fat-1*	Wild-type	↑	↑	↓	↑
**Hypertension**							
Mestre et al. (2018b)	Wild-type mice	Angiotensin-2	Vehicle	↓	–	–	–
Mortensen et al. (2019)	Hypertensive rats	*SHR*	Wild-type	↓	↓	–	–

Tracers refer to injected fluorescent/radiolabeled particles, including dextran, inulin, and/or ovalbumin, unless otherwise noted. The distribution of these tracers is generally taken as a surrogate marker for fluid movement within or between tissue compartments. Tracer influx denotes the movement of intracisternally injected molecules into brain tissue, while tracer efflux denotes the movement of intraparenchymally injected molecules out of brain tissue. The results of fluorescent/radiolabeled Aβ distribution studies are reported in the adjacent columns.

*Decreased peri-arterial dextran efflux with partial compensatory increase in peri-venous efflux noted in aged Tg2576 mice.

**Fluorescently labeled recombinant ApoE proteins were infused into the CSF and distribution of isoforms was compared (not fluid movement).

*CCI, controlled cortical impact; MMI, multiple microinfarction; pDMCAO, permanent distal middle cerebral artery occlusion; SAH, subarachnoid hemorrhage; SHR, spontaneously hypertensive rats; TBI, traumatic brain injury; tMCAO, transient middle cerebral artery occlusion.*

The incidence of CAA increases with aging, affecting 12–100% of patients over the age of 80 (Jellinger, 2002). Interestingly, multiple animal studies also support reductions in perivascular transport in aging. The thickness of the cerebrovascular BM increases with aging, doubling in size by 24 months of age (Ceafalan et al., 2019). Hawkes et al. (2011) found reduced peri-arterial drainage in aged wild-type mice, and Hawkes et al. (2013) reported reduced peri-arterial Aβ_1__–__4__0_ drainage. Additionally, Kress et al. (2014) identified reduced para-arterial CSF influx in aged wild-type animals, as well as reduced ISF efflux and Aβ_1__–__4__0_ clearance. A recent paper by Da Mesquita et al. (2018) identified impaired meningeal lymphatic outflow in the aging brain as a contributor to reduced CSF influx and ISF efflux.

ApoE genotype is a heritable risk factor for the development of CAA (Yamada, 2002). ApoE isoforms may affect the interaction of Aβ to the BM components, including laminin which facilitates the clearance of Aβ (Thal et al., 2007; Zekonyte et al., 2016). In fact, ApoE-ε4/Aβ complex has been shown to exhibit reduced binding to laminin-511, which may increase the deposition of Aβ (Castillo et al., 2000; Zekonyte et al., 2016). Hawkes et al. (2012) found that humanized ApoE-ε4 mice by the targeted gene replacement exhibited lower levels of laminin and collagen IV in the BM, and increased deposition of intrahippocampally injected Aβ_1__–__4__0_ within the walls of leptomeningeal arteries. Finally, Achariyar et al. (2016) found that the para-arterial distribution of ApoE had an isoform specificity, showing that ApoE-ε4 distribution was reduced compared to ApoE-ε2 and ApoE-ε3. These studies point to ApoE/Aβ/laminin interactions as an important regulator of Aβ_1__–__4__0_ transport through the BM and PVS.

Cerebrovascular insults and CAA commonly co-occur, with one study reporting that nearly 70% of patients with severe CAA pathology exhibited evidence of either infarction or hemorrhage on autopsy (Jellinger, 2002). Wang et al. (2017) found that stroke increased focal CSF solute trapping within the area of infarction. Venkat et al. (2017) showed that multiple microinfarctions decreased Aquaporin-4 (AQP4) levels resulted in glymphatic dysfunction. Furthermore, Howe et al. (2018a, 2019) reported BM remodeling increased peri-infarct deposition of CSF Aβ_1__–__4__0_ in aged animals with stroke. These studies also show a consistent effect of acute stroke on inhibiting perivascular transport and promoting the sequestration of CSF solutes, including Aβ_1__–__4__0_, within infarcted brain regions.

Hyperlipidemia may also remodel the cerebrovascular BM through the reduction of collagen IV and other components (de Aquino et al., 2019), which has been shown to also impact perivascular transport pathways. Hawkes et al. (2015) found mice that were first exposed to a maternal diet high in saturated fats, and then subsequently fed a high-fat diet themselves, exhibited impaired perivascular drainage of Aβ_1__–__4__0_ in adulthood. Furthermore, Ren et al. (2017) found that fat-1 transgenic mice, which have higher circulating levels of beneficial polyunsaturated fatty acids, exhibited increased rates of both CSF influx and ISF efflux, and that supplementation with fish oil in wild-type mice had a protective effect against CSF Aβ_1__–__4__0_ induced injury.

Chronic hypertension causes cerebrovascular BM thickening which may impair perivascular transport mechanisms (Held et al., 2017). Mestre et al. (2018b) found that acute hypertension by angiotensin-2 treatment reduced the efficiency of peri-arterial CSF influx due to increased backflow of solutes within the PVS. The recent studies by Mortensen et al. (2019) and Xue et al. (2020) showed that decreased CSF influx in spontaneous hypertensive rats (SHRs), an animal model of chronic hypertension. In addition, Koundal et al. (2020) found reduced glymphatic flow rate and extent in stroke-prone SHRs. The detrimental role of chronic hypertension on perivascular Aβ clearance merits further study, as chronic increases in systemic blood pressure worsens Aβ deposition (Gentile et al., 2009; Carnevale et al., 2012), which may also produce additional changes in the BM in cerebral small vessels that could contribute to altered CSF flow dynamics.

## BM Remodeling Impairs Perivascular Solute Transport by Multiple Mechanisms

**FIGURE 3:**
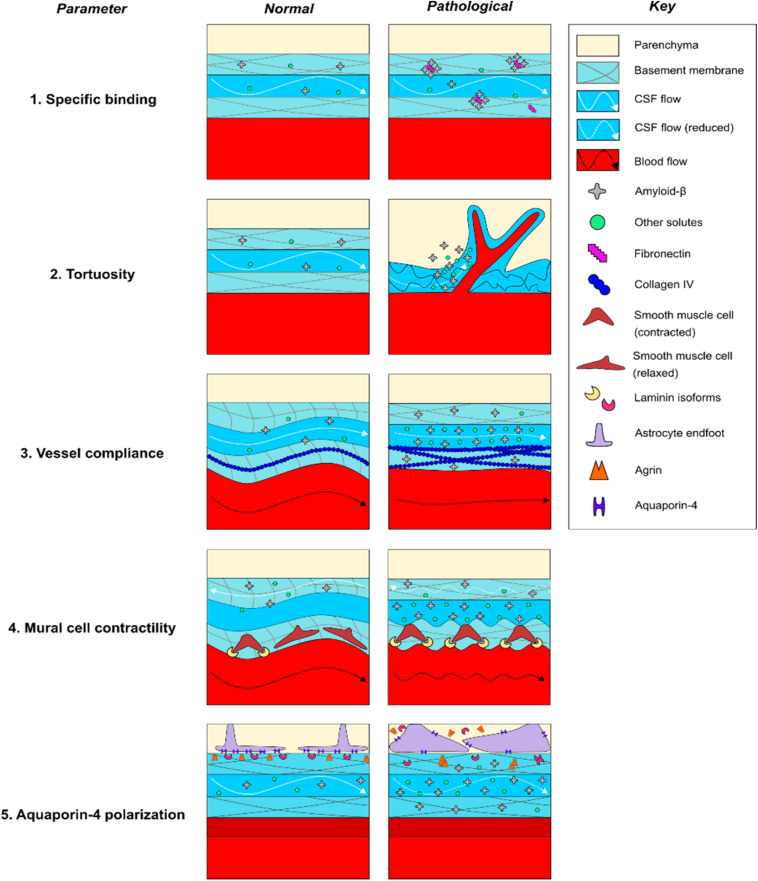
Potential mechanisms of perivascular transport impairment in CAA pathogenesis. (1) Increased expression of fibronectin may specifically favor Aβ deposition within the basement membrane, (2) alterations in the cellular and molecular geometry of the perivascular space may non-specifically trap Aβ and other solutes within areas of reduced flow, (3) increased collagen IV expression may reduce the compliance and alter the pulsatility of vessels, impairing paravascular flow through the PVS, (4) increased laminin expression may pathologically increase vasoconstriction of vessels and reduce peri-arterial BM drainage, and (5) altered laminin and agrin distribution may impair AQP4 polarization along astrocytic endfeet, reducing water permeability and impairing CSF-ISF exchange. Dashed lines indicate reduced rates of CSF flow.

In this section, we will summarize the evidence that BM remodeling contributes to the pathogenesis of CAA by impacting at least five distinct physiological parameters that have been demonstrated in the literature to modulate perivascular solute transport (Figure 3). BM remodeling may slow the perivascular transport of Aβ_1__–__4__0_ and promote CAA via multiple pathophysiologic mechanisms, including (1) specific binding of ECM proteins to soluble Aβ_1__–__4__0_, (2) increased tortuosity of the PVS to ISF solute diffusion, reduced vascular reactivity due to, (3) reduced vessel compliance, and (4) impaired contractility of mural cells, as well as (5) altered polarization of AQP4 leading to reduced CSF-ISF exchange. In an attempt to provide a clearer picture of the pathogenesis of CAA, this section will review each of these potential contributions to impaired perivascular flow, with an emphasis on the importance of BM remodeling in driving these processes.

### BM Remodeling May Increase Specific Binding of Amyloid Species

BM remodeling may favor amyloid deposition due to changes in BM protein composition that promote the binding of BM proteins to amyloid species (Figure 3). CAA is associated with a number of changes in BM composition that may favor the binding of amyloid species to the remodeled BM (Table 2). Neuropathological studies in human AD patients consistently report colocalization of amyloid plaques with abnormal deposits of BM components in the vicinity of brain microvessels; amyloid plaques have been shown to colocalize with staining for HSPGs (Snow et al., 1988; Perlmutter et al., 1991), agrin (Verbeek et al., 1999), perlecan (Lepelletier et al., 2017), collagen IV (Perlmutter et al., 1991; Lepelletier et al., 2017), laminin (Perlmutter et al., 1991), and fibronectin (De Jager et al., 2013; Lepelletier et al., 2017), although colocalization of plaques with fibronectin and perlecan has not always been observed (Koike et al., 1988; Verbeek et al., 1999). Moreover, multiple biochemical studies using C-truncated APP have confirmed specific roles for each of these BM proteins in binding to amyloid species *in vitro* (Narindrasorasak et al., 1991, 1992, 1995). It has also been shown that perlecan and laminin directly bind Aβ (Snow et al., 1995; Castillo et al., 2000). Recent work by our laboratory elucidated a potential pathophysiologic relationship between elevated fibronectin levels and Aβ_1__–__4__0_ deposition *in vivo*, finding that intracisternally infused CSF Aβ_1__–__4__0_ deposited within thickened, fibronectin-rich segments of the BM in aged mice with stroke, and that fibronectin-Aβ_1__–__4__0_ conjugation increased its deposition within the BM of pial vessels (Howe et al., 2018a). Taken together, these studies indicate that deposits containing fibronectin and other BM proteins may create a “trap” for Aβ_1__–__4__0_ circulating through the PVS due to specific molecular interactions. However, such binding does not always favor aggregation as laminin binding may inhibit Aβ fibril formation (Castillo et al., 2000). Future work in both wild-type and transgenic models are needed to confirm that changes in BM composition lead to amyloid plaque formation in CAA by a specific binding mechanism.

### BM Remodeling May Increase the Tortuosity of the PVS

BM remodeling may also increase the overall tortuosity of the PVS via multiple mechanisms (Figure 3). Tortuosity is an experimentally determined variable that reflects the rate of distribution of ISF solutes through the brain ECS, and it has been shown to increase in a variety of pathological states (Syková and Nicholson, 2008). Tortuosity has been shown to correlate with increased geometric path length around cells, increased dead spaces between cells, alterations in the electrical charge of the ECS, and general increases in viscosity due to weak interactions (Syková and Nicholson, 2008). In addition to their impact on diffusional forces, recent mathematical modeling indicates that arterial processes in the PVS are near-optimally located to reduce the hydraulic resistance resulting in an efficient bulk flow (Tithof et al., 2019), although the relative contribution of free diffusion and convective flux of ISF containing parenchymal solutes on their transport remains elusive, and has not been directly confirmed in experimental studies (Nicholson and Hrabìtová, 2017; Faghih and Sharp, 2018). With that said, there is evidence that the projection of cerebral vasculature is altered in CAA, including increased branching of microvessels and regional changes in capillary density. These changes could modify ECS geometry and increase the transit time of solutes contained within the BM, contributing to impairment of solute diffusion and bulk flow (Perlmutter et al., 1990; Yamaguchi et al., 1992; Shimizu et al., 2009; Hawkes et al., 2011; Lepelletier et al., 2017; Hase et al., 2019). One recent experimental study examined the impact of CAA on the geometry of brain microvessels using AD-model TgCRND8 mice. The study found that cortical arterioles exhibited increased branching and decreased diameter with amyloid pathology, which was associated with higher dispersion and elongation of microvascular network transit times of intravenous fluorescent tracers, although perivascular fluid movement through the brain ECS was not measured (Dorr et al., 2012). More directly examining changes in the diffusional properties of the brain ECS in AD-model APP23 mice, Syková et al. (2005) found that aged APP23 mice exhibited increased amyloid pathology, increased ECS volume fraction and decreased apparent diffusion coefficients compared to young controls, all of which is consistent with increased tortuosity (Syková and Nicholson, 2008). More work is needed to determine the impact that increased tortuosity has on the perivascular transport of soluble Aβ.

During CAA pathogenesis, BM remodeling may contribute to these observed increases in tortuosity. Regions of BM thickening have been shown to contain large deposits of collagen IV and HSPGs in human subjects with AD (Perlmutter et al., 1990; Claudio, 1995). Abnormal deposition of these ECM components has been hypothesized to increase tortuosity, but data examining the relationship between specific BM constituents and tortuosity in CAA are limited (Syková and Nicholson, 2008; Nicholson and Hrabìtová, 2017). In a study of human astrocytic tumors, Zámecník et al. (2004) compared ECM composition with tumor grade and then measured the tortuosity of the tissue using tetramethylammonium iontophoresis. They reported that increases in tortuosity positively correlated expression of ECM components, including type IV collagen, laminin, and fibronectin, that localized to the BM. While this study is consistent with the hypothesis that BM remodeling may lead to reduced solute diffusion, further work is needed to assess these relationships in CAA. There is also a need to establish the degree to which solute movement through the BM as well as through the parenchymal ECS is dependent on free diffusion and convective bulk flow, respectively, and whether these change in disease states.

### BM Remodeling May Diminish Pulsation Due to Reduced Vessel Compliance

BM remodeling may also act to reduce bulk flow by disrupting the pulsation of brain microvessels (Figure 3). Under normal conditions, the ability of pulsatile forces to propagate from the aortic root to the small vessels of the brain is dependent on the compliance of the vascular tree. During aging, reduced compliance of the peripheral vasculature transmits increased pulsatile forces to the cerebral vasculature, which is hypothesized to cause compensatory vasoconstriction and fibrosis that reduces the compliance and pulsation of small vessels (Mitchell et al., 2011; Muhire et al., 2019). The following several studies have examined the association of cerebrovascular compliance with cognitive function and imaging biomarkers of CAA in human subjects, including white matter hyperintensities and dilation of PVSs. Webb et al. (2012) found that white matter hyperintensities are associated with increased pulsatility of the middle cerebral artery, however, the specific impact on small vessel compliance was not examined. Subsequently, Cooper et al. (2016) found that aortic stiffness was associated with increased resistance of intraparenchymal vessels, white matter hyperintensities, and cognitive impairment. Thomas et al. (2019) found that increased aortic stiffness correlated with dilation of PVSs and decreased small vessel compliance in elderly individuals; cognitive status was not assessed. Finally, Rajna et al. (2019) found that cardiac pulse propagation to the CSF was significantly altered in the lateral ventricles of AD patients, characterized by reduced entropy and increased variance compared to age-matched controls, providing a potential link between reduced pulsation and impairment of CSF flow in human subjects. Overall, these studies point to changes in the pulsation of cerebral vessels in patients with CAA, but more work is needed that specifically examines changes in small vessel pulsatility.

There are limited data available on how brain microvessels respond to increased pulsatility of proximal cerebral arteries, however, there is some evidence that BM remodeling can reduce the compliance of brain microvessels. The majority of studies in AD patients and animal models have identified elevated BM collagen IV levels in the brain (Table 2). Studies in collagen IV mutant mice found that loss-of-function mutations increased vessel compliance, leading to distended vessels that are prone to hemorrhage in the perinatal period (Jeanne et al., 2015; Magaki et al., 2018; Ratelade et al., 2018; Hase et al., 2019). Further work in TgAPP mice, an animal model of AD, found increased collagen IV expression in the aorta was associated with reduced aortic responses to pharmacologic treatment with vasodilators (Navarro-Dorado et al., 2016). While the study did report increased collagen IV surrounding brain microvessels, direct physiologic measurements of brain microvessel compliance were not reported in this study. Finally, Iliff et al. (2013b) examined the relationship between the pulsation of intracortical penetrating arterioles and CSF-ISF exchange of fluorescent dextran with two-photon microscopy in anesthetized wild-type mice. The study reported that unilateral carotid ligation reduced the pulsatility of arterioles and limited CSF-ISF exchange, while conversely, acute treatment with the β_1_-adrenergic agonist dobutamine increased pulsatility and enhanced CSF-ISF exchange. While compliance was not directly measured, this study provides an important link between altered cerebrovascular pulsation and impairment of CSF-ISF exchange, which may also contribute to CAA pathogenesis. Overall, preclinical work suggests an important role for changes in cerebrovascular pulsation in modulating perivascular CSF-ISF exchange in animal models. While evidence suggests that vessel compliance and pulsatility play an important role in facilitating CSF-ISF exchange, further experiments are required to better establish how increases in collagen IV impact CSF-ISF exchange of Aβ in various stages of CAA pathogenesis.

### BM Remodeling May Impair Mural Cell Function and Vasomotion-Assisted Transport Mechanisms

There is emerging evidence showing the activity of mural cells (including pericytes and SMCs), could play an important role in facilitating the transport of solutes through both the PVS and the BM (Schley et al., 2006; Coloma et al., 2016; van Veluw et al., 2019; Figure 3). Mathematical modeling suggests that intrinsic vasomotion produces sufficient reflected waves to promote peri-arterial drainage of solutes through the normal BM (Schley et al., 2006; Coloma et al., 2016). During CAA pathogenesis, reduced intrinsic vasomotion of arteries and arterioles might occur in relation to vascular damage (Kisler et al., 2017). These Aβ-related changes may include degeneration of mural cells, which contributes to constriction, hypoperfusion, and BBB breakdown (Kisler et al., 2017), as seen in impaired responses in the neurons and the vasculature in CAA patients (Dumas et al., 2012; Peca et al., 2013; Williams et al., 2017) and in animal models (van Veluw et al., 2019). This is evidenced by Dumas et al. (2012), which found that CAA patients exhibited impaired BOLD responses to visual stimulation, characterized by reduced response amplitude, prolonged time to peak, and prolonged time to baseline, which correlated with white matter hyperintensities and is consistent with impaired neurovascular coupling. Peca et al. (2013) found similar reductions in occipital cortical responses to repetitive visual stimulation in CAA patients, which also correlated with increased white matter hyperintensities and microbleeds. Williams et al. (2017) specifically examined hemodynamic response function (HRF), an integrated measure of neurovascular coupling, and found that multiple parameters of the HRF were associated with CAA and microbleeds. Importantly, emerging research suggests that both neuronal and vascular activities influence perivascular clearance. In a recent study, van Veluw et al. (2019) examined the effect of visual stimulation on the paravascular solute clearance in wild-type and APP/PS1 mice. In this study, fluorescent dextran was first introduced into the PVS of awake animals by the focal laser ablation of a blood vessel, who were subsequently exposed to repetitive visual stimulation. The study identified reduced vascular reactivity in mice with CAA, which correlated with reductions in paravascular clearance and loss of SMC coverage. In summary, the above studies suggest that neurovascular coupling is impaired in both animal models and human subjects with CAA, and play a potentially significant role in modulating perivascular waste clearance.

Reduced cerebrovascular reactivity in CAA may be related to decreased pericyte (Giannoni et al., 2016; Halliday et al., 2016) and SMC (Tian et al., 2006; Miners et al., 2011; Han et al., 2015; Keable et al., 2016; van Veluw et al., 2019) coverage. Multiple studies support a role for the healthy BM in supporting mural cell development, survival, and function under normal conditions. Chen et al. (2013) examined SMC function in astrocyte-specific laminin knockout mice, and found that SMC differentiation and the expression of contractile proteins were reduced in the knockout animals, leading to vasodilation and hemorrhagic stroke (Chen et al., 2013). In contrast, a subsequent study in pericytes found that inhibition of astrocytic laminin-111 binding to integrin α2 increased pericyte differentiation and contractile protein expression (Yao et al., 2014). Finally, Merlini et al. (2011) found that CAA pathology in AD-model Tg-ArcAβ mice was associated with laminin overexpression, SMC loss, and neuropathological signs of neurovascular decoupling. Laminin expression was found to be highest in vessels impacted by CAA, further suggesting a potential negative effect of laminin on SMC function (Merlini et al., 2011). Taken together, these studies suggest that increased levels of laminin may reduce vasomotion during CAA pathogenesis, although more work is needed to decipher potentially divergent effects of laminin on pericyte and SMC activity and survival, respectively.

### BM Remodeling May Impair Perivascular AQP4 Polarization

BM remodeling may also influence para-vascular amyloid transport via indirect effects on AQP4 (Mestre et al., 2018a), a water channel that is expressed on astrocyte endfeet (Figure 3). In healthy brain tissue, AQP4 is polarized to the BM-facing surface of astrocytic endfeet (Nakada et al., 2017). Knockout of AQP4 has been shown to inhibit both CSF influx and para-venous efflux in animal models, leading to a reduction in the clearance of Aβ_1__–__4__0_ from the brain parenchyma (Iliff et al., 2012). Multiple studies report increased astrocyte reactivity and impaired AQP4 polarization in CAA (Wilcock et al., 2009; Merlini et al., 2011; Zeppenfeld et al., 2017; Boespflug et al., 2018). Yang et al. (2017) found that AQP4 polarization is decreased in AD-model Tg-ArcSwe mice, in part, due to increased expression levels on reactive astrocytes surrounding amyloid plaques. Furthermore, Xu et al. (2015) reported that AQP4 deletion worsened CAA pathology and cognitive deficits in APP/PS1 mice. Zeppenfeld et al. (2017) showed that the degree of AQP4 polarization inversely correlated with amyloid burden in AD brains, and individuals with cognitive impairment had lower levels of AQP4 polarization than age-matched controls on autopsy. Burfeind et al. (2017) identified four single nucleotide polymorphisms in the AQP4 gene that were associated with altered rates of cognitive decline in AD patients. These findings support an important role for dysregulated astrocytic responses and altered AQP4 polarization in CAA.

BM remodeling may impair AQP4 polarization due to altered receptor binding at the astrocytic endfoot. The healthy BM regulates the expression of AQP4. Studies indicate a significant role for multiple BM proteins, including laminin isoforms 111 and 211, as well as agrin and astrocytic β_1__–_integrin in maintaining AQP4 polarization (Noell et al., 2009; Robel et al., 2009; Fallier-Becker et al., 2011; Menezes et al., 2014; Yao et al., 2014; Gautam et al., 2016; Noël et al., 2019, 2020). Laminins have been shown to promote AQP4 polarization via binding to the dystroglycan associated complex on the cell membrane (Noël et al., 2020), which has been shown to bind AQP4 via α-syntrophin and locally modulate its expression on astrocytic endfeet (Noell et al., 2011; Tham et al., 2016). Interestingly, this process may be disrupted in CAA, as recent transcriptional network analysis of human astrocytic endfoot genes showed reduced levels of dystroglycan, dystrobrevin, and α-syntrophin in AD patients (Simon et al., 2018). In addition to disruption of the dystroglycan pathway, abnormal focal deposits of agrin and laminin could promote the mislocalization of AQP4 to locations outside of the PVS, reducing the polarization of AQP4 to astrocyte endfeet (Noell et al., 2009; Fallier-Becker et al., 2011; Menezes et al., 2014; Yao et al., 2014; Gautam et al., 2016; Noël et al., 2019; Table 2). This provides an important link between alterations in BM components, loss of AQP4 polarization and impaired perivascular transport of Aβ_1__–__4__0_ observed in CAA.

## Discussion and Clinical Significance

The present review of the literature supports the hypothesis that BM remodeling contributes to the pathogenesis of CAA, in part, by altering the perivascular transport of soluble Aβ_1__–__4__0_ from the brain. Perivascular transport is mediated by a complex microenvironment that requires the concerted activity of multiple cell types to maintain effective transport of waste products. BM remodeling, characterized by changes in morphology and composition, occurs early in CAA development and may be an important driver of Aβ deposition due to its effects on perivascular transport pathways relevant to Aβ_1__–__4__0_ clearance. The BM provides an important route for perivascular transport, which occurs along multiple pathways, including para-vascular influx and efflux through the PVS, as well as peri-arterial influx and drainage along with the glial/pial BM (influx) and smooth muscle BM (efflux), respectively (Albargothy et al., 2018). Each of these processes may be disrupted in CAA as well as related risk factors and provides a potential explanation for the perivascular distribution of amyloid aggregates. Finally, emerging research indicates that BM remodeling produces changes in multiple parameters relevant to perivascular transport, including increased Aβ affinity, increased tortuosity, altered pulsatility, impaired vasomotor responses, and decreased water permeability.

In addition to perivascular transport, there are numerous other factors that also play a role in CAA pathogenesis that are beyond the scope of this review. Neurodegeneration may be associated with increased APP metabolism and production of Aβ isoforms by neurons and endothelial cells, which is thought to contribute to perivascular amyloid plaques (Kalaria et al., 1996; Bhadbhade and Cheng, 2012). Additionally, microglia and other immune cells have been shown to provoke a neuroinflammatory response and assist with the clearance of amyloid plaques via phagocytosis (Fang et al., 2010; Tarasoff-Conway et al., 2015). Furthermore, alterations in the intracellular degradation of Aβ_1__–__4__0_ by endo-lysosomal compartments, which involves the ubiquitin-E3 ligase pathway, may also be affected in AD patients (Tarasoff-Conway et al., 2015; Harris et al., 2020). Alternatively, Aβ may be transported between the ISF and the systemic circulation by transendothelial active transport, assisted by various mediators including p-glycoprotein 1, LRP1, RAGE, alpha2-macroglobulin, clusterin, and apolipoprotein J (Tarasoff-Conway et al., 2015; Reed et al., 2019). Finally, multiple mutations in the APP gene that increase misfolding and aggregation can predispose individuals to develop hereditary cerebral hemorrhage with amyloidosis (HCHWA), a heritable form of CAA that is associated with early and severe disease onset (Luyendijk et al., 1988; Van Broeckhoven et al., 1990; van Nostrand et al., 1992, 2001; Davis et al., 2004). These factors contribute to disease pathogenesis and have the potential to be integrated into the perivascular transport hypothesis with further study.

The perivascular transport mechanisms have the potential to revolutionize the prevention and early detection of CAA pathology. A recent meta-analysis found that the CSF levels of Aβ_1__–__4__0_ and Aβ_1__–__4__2_ were significantly lower in symptomatic, sporadic CAA patients compared to healthy controls, which could be related to impaired perivascular clearance or increased re-circulation of waste products along perivascular transport pathways (Charidimou et al., 2018). Furthermore, in a recent study of HCHWA patients, young pre-symptomatic carriers were found to exhibit significantly lower levels of CSF Aβ_1__–__4__0_ and Aβ_1__–__4__2_ compared to age-matched non-carriers, raising the possibility that declining perivascular Aβ transport could occur decades before the development of symptomatic CAA in affected individuals (van Etten et al., 2017). In addition to direct measurement of CSF proteins, the rapid development of new imaging modalities may soon provide more direct biomarkers of impairment in perivascular transport, potentially improving the detection of pre-symptomatic disease in human patients (Taoka et al., 2017; Ringstad et al., 2018; Rajna et al., 2019). Still more techniques could be used to measure BM remodeling, with specific PET imaging ligands available that provide biomarkers of fibrosis (Beziere et al., 2019), AQP4 levels (Nakamura et al., 2011), and reactive astrogliosis (Cavaliere et al., 2020) that may precede impairment in perivascular transport. Even blood biomarkers could prove useful in diagnosing CAA, as our laboratory has identified serum markers of BM remodeling that may distinguish between CAA and hypertensive intracerebral hemorrhage etiologies (Howe et al., 2018b). Overall, the perivascular transport process may provide a useful model with which to develop novel biomarkers of CAA. When identified, these new biomarkers may help to reduce the burden of dementia in vulnerable populations by identifying patients prior to the development of significant amyloid pathology.

The perivascular transport mechanisms also suggest several therapeutic avenues that may be used to prevent or slow cognitive decline in the elderly. Numerous clinical trials have tested anti-amyloid therapies in human dementia patients, which have shown little improvement in cognitive function despite significant reductions in total brain amyloid levels (van Dyck, 2018). This suggests that targeting amyloid plaques directly may be a suboptimal treatment strategy for age-related dementia. Reversing BM remodeling and boosting perivascular transport via lifestyle modification or pharmacologic treatment could provide an alternative strategy for treating dementia before amyloid accumulation occurs. For example, studies have found that exercise is protective against dementia in humans (Karssemeijer et al., 2017), which may be partially explained by improvements in perivascular transport processes (He et al., 2017; von Holstein-Rathlou et al., 2018). Similarly, treatment with fish oil has been shown to protect against dementia in humans (Zhang et al., 2016), and supplementation has also been shown to boost perivascular transport and protect against CSF amyloid-induced neuroinflammation in mice (Ren et al., 2017). Staying mentally active has been shown to stave off dementia in humans (Bahar-Fuchs et al., 2019), which could be related to neurovascular coupling and perivascular transport (van Veluw et al., 2019). Finally, cerebrovascular disease is a major driver of cognitive impairment in the elderly (Ivan et al., 2004; Mahon et al., 2017), and recently published work by our laboratory found that treatment with a transforming growth factor-β (TGF-β) receptor antagonist reversed BM remodeling and improved perivascular CSF influx in an aged mouse model of stroke, providing a potential tool to mitigate the negative effects of cerebrovascular disease on perivascular transport (Howe et al., 2019). Overall, the above studies showcase the ability of these perivascular transport mechanisms to explain the benefits of lifestyle modification in slowing age-related cognitive decline, as well as identify new potential therapeutic avenues that may prove useful in the primary and secondary prevention of dementia.

In conclusion, the impact of BM remodeling on perivascular transport pathways remains an area of highly active research, with significant potential for improving our understanding of the pathogenesis of CAA. It also provides an important link between cardiovascular risk factors and amyloid accumulation in age-related dementia. While this model represents just a single facet of a complex disease process, it holds promise in guiding future research in two key areas: (1) early detection of individuals who are at-risk of developing CAA, as well as, (2) the development of potential treatments that modify disease-associated BM remodeling before significant neurodegeneration occurs. Accomplishing these two goals could significantly reduce the burden of CAA on our society, and provide improved quality of life to our aging population.

## Author Contributions

MH and AU conceptualized the review topic and managed the peer review process. MH, LM, and AU wrote the manuscript. All authors contributed to the article and approved the submitted version.

## Conflict of Interest

The authors declare that the research was conducted in the absence of any commercial or financial relationships that could be construed as a potential conflict of interest.
